# COVID-19 disruption: Family physicians at the forefront

**DOI:** 10.4102/safp.v63i1.5286

**Published:** 2021-03-10

**Authors:** Johannes F. Hugo

**Affiliations:** 1 Department of Family Medicine, Faculty of Health Sciences, University of Pretoria, Pretoria, South Africa; 2 Community Oriented Primary Care Research Unit (COPC), University of Pretoria, Pretoria, South Africa

In our wildest nightmares, we would not have dreamt up coronavirus disease 2019 (COVID-19). It disrupted our world beyond recognition. Let us first remember and honour our colleagues who have lost the battle against COVID-19 and those who have lost family, friends and colleagues. And let us think about those who are recovering.

That brings us to the first issue: COVID-19 poses a real threat to the health of health workers, and primary care providers (including general practitioners, medical officers, family physicians, nurse clinicians, clinical associates) have all been in a particularly difficult situation as they provide first-contact care. Asymptomatic transmission, early disease and stigma (causing people to hide their symptoms) have all made it more difficult to stay safe in primary care.

Family physicians have engaged strongly with the pandemic in various ways; especially by building networks on local, provincial, national and international platforms. These networks have ranged from building a local network of lead clinicians in the district to building a World Organization of Family Doctors (WONCA) Africa network of webinars to address COVID-19.

Some have taken on the challenge of caring for people with chronic diseases through telemedicine and home delivery of medicine.^1^ Others have become engaged with restructuring health service care at clinics and field hospitals, even changing a convention centre into a COVID-19 field hospital.^2,3^ Still others have become engaged on short notice with the care of homeless people in shelters, providing health services and substance use services at large scale and changing the approach to homeless people in big cities.^4^

A family medicine department is implementing a huge project in partnership with an international mining company, developing care for mine workers and mining communities through a community-oriented primary care (COPC) intervention. This may pave the way for a new approach to healthcare and development in mining communities.^5^

The personal protective equipment (PPE) corruption debacle had a significant impact on senior family physicians and other senior clinicians. Whilst they worked very hard to develop and propose interventions in primary care, the people who were supposed to work on budgets and allocations were busy working on schemes to raid the funds meant for COVID-19 response. In my own experience, none of the proposals we made came to fruition. Whilst the basic task of leadership (politicians, senior administrators and senior clinicians) is to negotiate the use of resources to serve the people, what we observed was negotiating resources to enrich the elite. This will clearly stay a challenge for the future. We will have to be much more aware of what is happening and be vigilant in demanding transparency. We will have to be prepared to speak truth to power to protect the health of society.

In an article about successful responses to COVID-19 from complexity theory, Begun and Jiang wrote that the best responses were built on communication, collaboration and innovation done at speed.^6^ This requires strong leadership from clinicians and administrators. This is exactly what family physicians are doing, and more of this will be needed in future.

Coronavirus disease 2019 is far from over; the situation is complexifying, and the uncertainties are growing. And we can expect more epidemics and pandemics. Family physicians will always be at the forefront, and we will have to work out ways to address these. Developing an integrated community-based healthcare system is one way to prepare for and manage epidemics. And we will have to take the lead and work hard through service delivery, research, education and advocacy to face the future.
